# Targeting steroid receptor RNA activator (SRA), a long non-coding RNA, enhances melanogenesis through activation of TRP1 and inhibition of p38 phosphorylation

**DOI:** 10.1371/journal.pone.0237577

**Published:** 2020-08-13

**Authors:** Ji-Chen Ho, Chih-Hung Lee, Chien-Hui Hong

**Affiliations:** 1 Department of Dermatology, Kaohsiung Chang Gung Memorial Hospital and Chang Gung University College of Medicine, Kaohsiung, Taiwan; 2 Department of Dermatology, Chiayi Chang Gung Memorial Hospital, Chiayi, Taiwan; 3 Department of Dermatology, National Yang-Ming University, Taipei, Taiwan; 4 Department of Dermatology, Kaohsiung Veterans General Hospital, Kaohsiung, Taiwan; University of Florida, UNITED STATES

## Abstract

Abnormal skin melanin homeostasis results in refractory pigmentary diseases. Melanogenesis is influenced by gene regulation, ultraviolet radiation, and host epigenetic responses. Steroid receptor RNA activator (SRA), a long noncoding RNA, is known to regulate steroidogenesis and tumorigenesis. However, how SRA contributes to melanogenesis remains unknown. Using RNA interference against SRA in B16 and A375 melanoma cells, we observed increased pigmentation and increased expression of TRP1 and TRP2 at transcriptional and translational levels only in B16 cells. The constitutive phosphorylation of p38 in B16-shCtrl cells was inhibited in cells with knocked down SRAi. Moreover, the melanin content of control B16 cells was increased by SB202190, a p38 inhibitor. Furthermore, reduced p38 phosphorylation, enhanced TRP1 expression, and hypermelanosis were observed in A375 cells with RNA interference. These results indicate that SRA-p38-TRP1 axis has a regulatory role in melanin homeostasis and that SRA might be a potential therapeutic target for treating pigmentary diseases.

## Introduction

Melanin synthesis is governed by several enzymatic processes, including the hydroxylation of L-tyrosine into L-dopa, which is a rate-limiting step, by the enzyme tyrosinase [1]. L-dopa is then oxidized to O-quinone. Tyrosinase-related protein (TRP)1 and TRP2 catalyze the conversion of dopachrome to melanin through several intermediates. The expression of tyrosinase, TRP1, and TRP2 are regulated by Microphthalmia-associated transcription factor (MITF), an important transcription factor in melanin synthesis. Other than MITF, melanocyte-specific melanocortin-1 receptor (MC1R) and its ligand, α-melanocyte stimulating hormone (α-MSH), as well as stem cell factor (SCF)/c-KIT receptor tyrosinase pathway both actively regulate melanogenesis. Abnormal melanin synthesis through signaling pathways such as extracellular-signal-regulated kinase (ERK), Adenosine 3',5'-cyclic monophosphate (cAMP), and c-Jun N-terminal kinase (JNK), results in several pigmentary diseases such as melasma, vitiligo, and graying of hair.

In addition to MITF, several other endogenous and exogenous mediators are involved in melanogenesis. Since melanin synthesis is regulated at least partially by exogenous factors, such as ultraviolet radiation, it is likely that epigenetic regulation such as DNA methylation, histone modification, and miRNA changes might influence melanogenesis. For example, 5-azacytidine, a DNA methylation inhibitor, decreases melanin synthesis by downregulating MITF and tyrosinase via cAMP response element-binding protein (CREB) inactivation [2]. Previous studies have demonstrated that several non-coding RNAs, such as miRNAs, can regulate the process of melanin synthesis. For example, miR-145 has been identified as one of the key regulators in melanogenesis [3]. The expression of miR‐675was reduced in the hyperpigmented skin of melasma patients and it mediates melanogenesis through MITF [4].

Long non-coding RNAs (lncRNAs) have been documented to be involved in many physiological and pathological processes such as inflammation [5], oncogenesis [6], allergy, and immunity of infection [7]. In the development of melanoma, the dysregulation of a number of lncRNAs, such as HOTAIR, MALAT1, BANCR, ANRIL, SPRY-IT1, and SAMMSON, has been reported to be involved [6, 8, 9]. However, whether lncRNAs are involved in melanogenesis remains unexplored. UCA1, a lncRNA, negatively regulates the melanogenesis through inhibiting the cAMP/PKA, ERK, and JNK signaling pathways in melanocytes [10]. Zeng et al. showed that Lnc-CD1D-2:1, a lncRNA, is involved in the UVB-induced upregulation and activation of tyrosinase [11]. They also showed that TUG1 and UCA1, both of which are lncRNAs, negatively regulate melanogenesis via the ERK pathway [12,13].

We then investigated how other novel lncRNAs might be involved in melanogenesis and melanoma progression. We have previously found that steroid receptor RNA activator (SRA) is consistently upregulated in several melanoma cell lines, including B16 cells, and SRA has a functional role in melanoma progression [14]. A PUBMED search with the keywords “steroid receptor RNA activator” returned only 102 results as of May 2020. In 2016, Liu et al. reviewed the role of SRA in several physiological processes, including myogenesis and steroidogenesis. In addition, SRA was also found to contribute to several pathological processes, including obesity, tumorigenesis, and cardiomyopathy [15]. Specifically in the process of tumorigenesis, SRA was reported to be actively involved in the progression of benign tumors and malignant cancers that are related to steroid or sex hormones, including ovarian endometriosis [16], polycystic ovary [17], uterine myoma [18], as well as endocrine cancers, breast cancers, ovarian cancers [19], and cervical cancers [20]. Because some pigmentary diseases have gender differences, for example, melasma preferentially affects woman, we remain interested whether SRA, which helps regulate cellular pathways related to steroid or sex hormone, regulates melanogenesis in melanocytes. In this study, we aimed to determine how SRA regulates melanogenesis in melanocytes by measuring the concentration of melanin and melanogenic enzymes upon the RNA interference against SRA.

## Materials and methods

### shRNA transfection in melanoma cells

A375 and B16 melanoma cell lines were acquired from ATCC. SRA silencing was achieved through the transfection of B16 cells with SRA shRNA plasmid (NM025291) or pLKO.1-puro empty vector control plasmid (SHC001, Sigma-Aldrich). Cells were seeded into 6-well plates with 5 ×10^4^ cells/well and transfected with 40 nM shRNA by using Lipofectamine 3000 (Invitrogen) according to the manufacturer's instructions. The target sequence for shSRA was 5′-CAGACTCACTTCACCTGACTT-3′. Cells were selected by using 2 μg/ml puromycin (Sigma-Aldrich) based on the killing assay.

### Real-time PCR

Total RNA was extracted by using the RNeasy Mini kit (#74106, QIAGEN) according to the manufacturer's instructions. One microgram of total RNA was used for cDNA synthesis by using PrimeScript RT Reagent Kit (RR037A, TaKaRa). Real-time PCR was performed by using an ABI PRISM 7500 Sequence Detection System (Applied Biosystems). The geometric mean concentration of the housekeeping gene β-actin was used as an internal control for the standardization of the expression levels and analyzed using the 2^–ΔCt^ method. The primers used and their corresponding target genes are as follows (Table 1).

**Table 1:** The primers for the SRA, MITF, TYR, TRP1, TRP2, MC1R, and β-actin.

Gene	Primer	Sequence
SRA	Forward	5′-CGCGGCTGGAACGACCCGCCGC-3’
	Reverse	5′-CAGACTCACCGGACACCATCTCCTA-3’
MITF	Forward	5′-GTGCAGACCCACCTGGAA AAC-3′
	Reverse	5′-AGT TAAGAGTGAGCATAGCCATAG-3′
TYR	Forward	5′-TCTGGACCTCAGTTCCCCTTC-3’
	Reverse	5′-AACTTACAGTTTCCGCAGTTGA-3’
TRP1	Forward	5′-CTGGATCAATGGATAGAACTGCC-3’
	Reverse	5′-CGACTGGCCTTGTTCCAAGT-3’
TRP2	Forward	5′-AGCAGACGGAACACTGGACT-3’
	Reverse	5’-CATCTGTGGAAGGGTTGTT-3’
MC1R	Forward	5’-TGGACAATGTCATTGACGTGATC-3’
	Reverse	5’-TGGTAGCGAGTGCGTAGAA -3’
β-actin	Forward	5’-GGCGGCACCACCATGTACCCT-3’
	Reverse	5’-AGGGGCCGGACTCGTCATACT-3’

### Western blot of p38, TRP1, TRP2, MITF, TYR, and NOTCH-1

Cells were seeded in a 6-cm dish. The DMEM culture medium was discarded, and cells were washed twice with phosphate-buffered saline (PBS) and subsequently lysed in 100 μl of 1x cell lysis buffer (#9803, Cell Signaling). The supernatant was collected and the sample buffer (#7722, Cell Signaling) was added. The mixture was subsequently denatured at 100°C for 10 min. Protein concentration was determined using a protein assay dye (#5000006, BIO-RAD), and proteins were electrophoresed by using precast protein gels 4–12% Bis-Tris Gel (NuPAGE, Thermo Fisher Scientific). The fractionated proteins in the protein gels were electrotransferred onto a nitrocellulose membrane (Millipore Co., Massachusetts). The membranes were blocked with 5% BSA in PBS at 25 ± 2°C for 1 h. After washing three times with PBS, the nitrocellulose membrane was incubated overnight with the primary antibodies diluted in 5% BSA: anti-β-actin antibody (MAB1501, Millipore) diluted at 1:10000, anti-MITF antibody (ab20663, Abcam) diluted at 1:1500, anti-TRP1 antibody (ab178676, Abcam) diluted at 1:10000, anti-TRP2 antibody (ab74073, Abcam) diluted 1:1000, anti-TYR antibody (ab180753, Abcam) diluted at 1:1000, anti-pp38 antibody (#9211, Cell Signaling) diluted at 1:1000, anti-p38 antibody (#9212, Cell Signaling Technology) diluted 1:1000, anti-Notch1 antibody (#2421, Cell Signaling Technology) diluted at 1:1000, and anti-cleaved Notch 1 (#4147, Cell Signaling) diluted at 1:1000. The excess primary antibodies were removed by washing the membranes three times with PBS, followed by incubation with anti-rabbit (AP132, Millipore) or anti-mouse (AP124, Millipore) secondary antibodies for 1 h at 20–25°C. The membrane was washed extensively in PBS with Tween™ 20 to remove any excess secondary antibody. Blots were visualized with Amersham ECL Prime Western Blotting Detection Reagent (RPN2106, GE Healthcare) and photographed with Syngene PXi4 Gel Documentation System.

### Tyrosinase activity

Cells (5 × 10^5^/mL) were washed twice with PBS. Cell pellets were subsequently mixed with 200 μl 50 mmol/L phosphate buffer (pH 6.8, #12940704, Thermo Fisher Scientific) containing 0.1% Triton X-100 for 10 min on ice (zero degree). The cell suspension was then centrifuged at 500 × g for 10 min, and the supernatant was collected into a new tube. The supernatant was then incubated with 50 μL of phosphate buffer (pH 6.8) containing 2 mM L-tyrosine for 5 h at 37°C in in the absence of light. Finally, a spectrophotometer was used to determine the absorbance at 490 nm.

### Measurement of melanin content

Cells were seeded into 6-cm dishes with approximately 5 × 10^5^ cells. After 24 h incubation at 37°C, the medium was changed and supplemented with DMSO with or without 10 μM SB202190 (S7067, Sigma) for another 24 h. Cells detached from the dishes by trypsinization and were counted using trypan blue. Cells were rendered soluble in 1N NaOH at 80°C for 1 h. Absorbances were then measured at 475 nm. Melanin content was determined against a standard curve of synthetic melanin (Sigma-Aldrich).

### Statistical analysis

The numerical variables between the two groups (for example, the densitometric data from western blot and the expression level of SRA between two groups) were compared by using Student’s t-test. The numerical variables among several groups were compared by using ANOVA with post-hoc comparison using Scheffe’s test. Statistical analysis was performed by using SPSS ver. 14 (Chicago, IL, USA). A *p*-value of less than 0.05 was considered statistically significant.

## Results

### SRA knockdown enhanced melanin pigmentation and TRP1 expression in B16 cells

**Fig 1:**
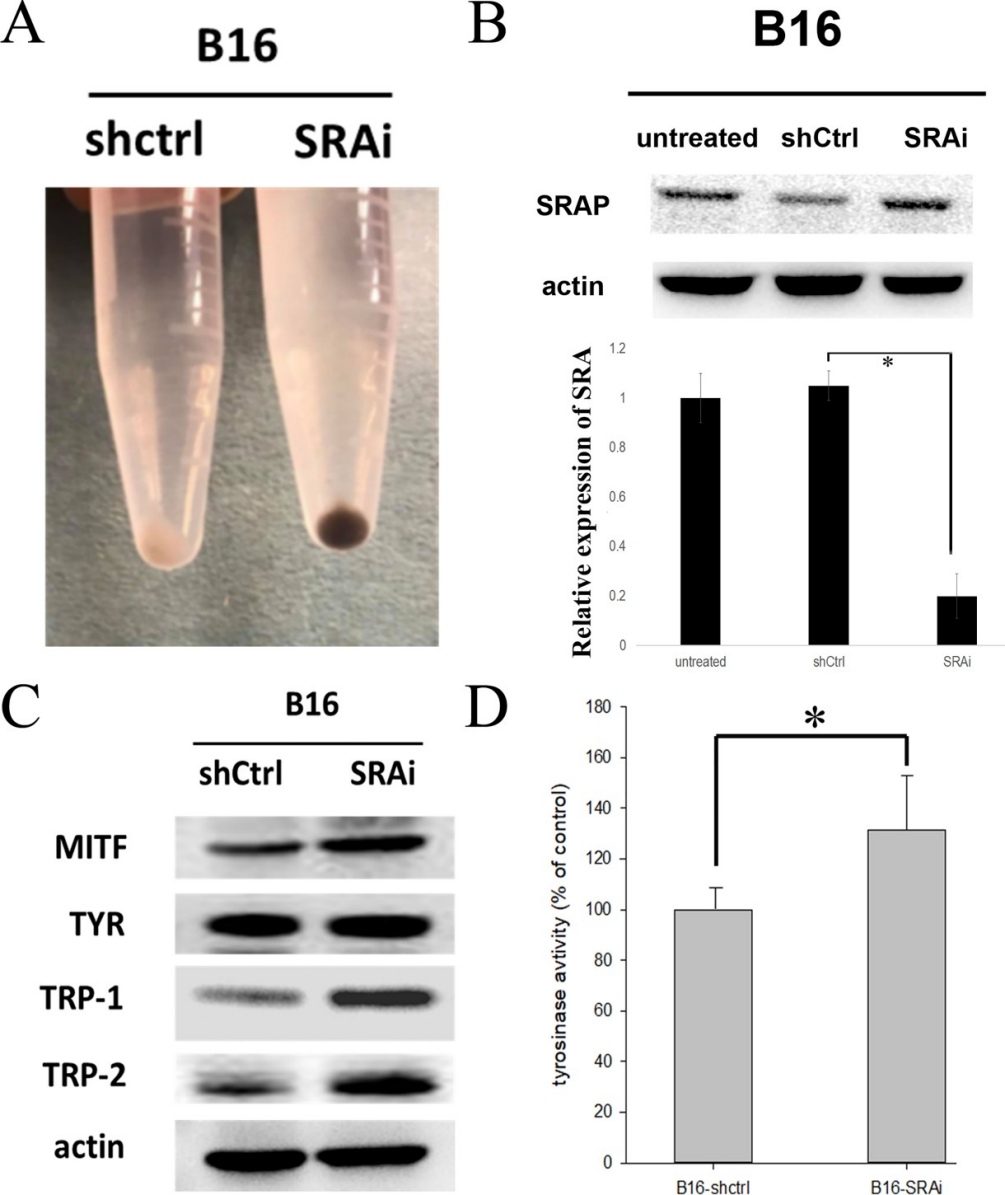
Increased pigmentation and enhanced TRP1 and TRP2 expression in SRA-deficient B16 cells and B16 cells were transfected with either siRNA against SRA (SRAi) or control sequence (siCtrl). (A) The gross pigmentation of B16-shCtrl cells and B16-SRAi cells. (B) Cells were evaluated for SRAP and SRA by western blotting (two repeated experiments with one representative blot) and by RTPCR (n = 3, error bars represent standard errors; * indicates p<0.05), respectively. (C) Cell lysates processed for western blotting to measure the expression of MITF, TYR, TRP1, and TRP2. Three repeated experiments with one representative blot. (D) Measurement and comparison of tyrosinase activity between B16-shCtrl cells and B16-SRAi cells (n = 3 and 3, respectively; error bars represent standard errors; * indicates p<0.05).

B16 melanoma cells with SRA deficiency showed a marked increase in pigmentation (Fig 1A). We then evaluated whether melanogenesis would be affected by SRA inhibition (Fig 1B). B16 cells were transfected with siRNA targeting SRA (SRAi) or control siRNA (shCtrl). While the expression of steroid receptor RNA activator protein (SRAP) was not different among the untransfected (untreated), shRNA-control (shCtrl), and shRNA-SRA (SRAi) cells, the expression of SRA was knocked down by 80% (Fig 1B). Moreover, to investigate the expression of melanogenesis enzymes when SRA was knocked down, we measured the expression of MITF, tyrosinase (TYR), TRP1, and TRP2 through western blot. Results showed that the expression of TRP1 in B16-SRAi cells was higher than that in B16-shCtrl cells, whereas the expression of MITF and TRP2 was only slightly higher in B16-SRAi cells than that in B16-shCtrl cells (Fig 1C). Although tyrosinase protein expression was not increased in B16-SRAi cells, its activity was increased by 20% in B16-SRAi cells (Fig 1D).

### Transcriptional levels of TRP1 and TRP2 were increased in B16-SRAi cells

**Fig 2:**
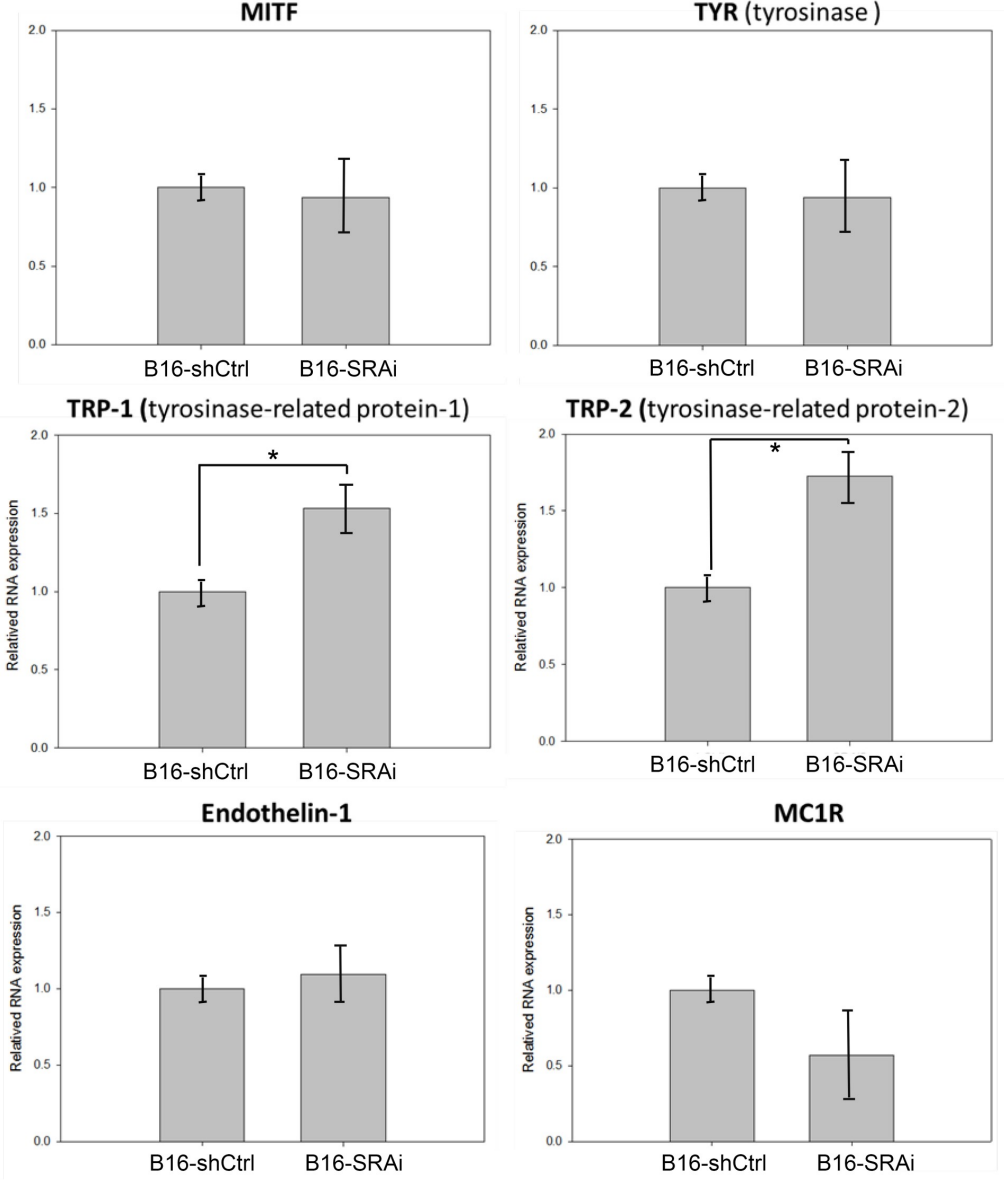
Increased TRP1 and TRP2 expressions in SRA-deficient B16 cells. B16-shCtrl and B16-SRAi cells were processed for RNA extraction, cDNA synthesis, and real-time PCR for the measurement of MITF, TYR, TRP1, TRP2, endothelin-1, and MC1R (n = 3 and 3, respectively; error bars represent standard errors; * indicates p<0.05).

We observed a marked increase in TRP1 concentration and a slight increase in the TRP2 and MITF concentration in B16-SRAi cells. We then validated the levels of these melanogenic enzymes at the transcriptional level by using real time PCR. The results showed that the expression levels of TRP1 and TRP2 were 50% higher in B16-SRAi cells than in B16-shCtrl cells, whereas, the transcriptional levels of TYR, MITF, and endothelin-1 did not differ between B16 cells with or without knocked down SRA (Fig 2). Although there seemed to be a 50% reduction in the MC1R expression of B16-SRAi cells compared with those of B16-shCtrl cells, the difference was not statistically significant (Fig 2).

### Enhanced p38 phosphorylation may mediate enhanced pigmentations in B16-SRAi cells

**Fig 3:**
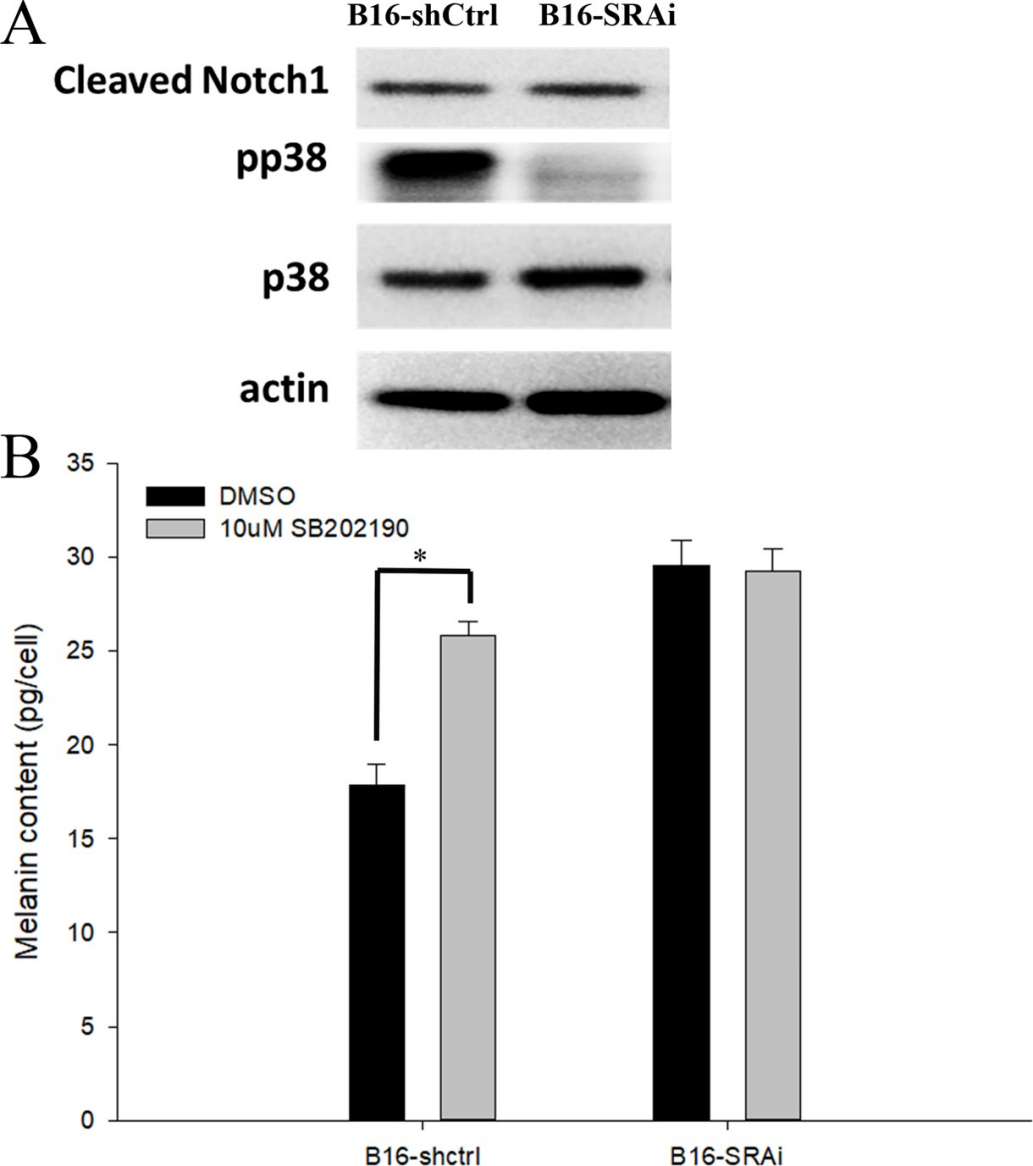
(A) Constitutive activation of p38 was obliterated in SRA-deficient B16 cells. The expression of total and phosphorylated p38 as well as cleaved Notch-1, were measured by western blot. Three repeated experiments with one representative blot. (B) Inhibition of p38 in control B16 cells (B16-shCtrl) enhanced pigmentations. SB202190, a p38 inhibitor, at 10 μM, was incubated with B16 cells for 24 h. Melanin concentration was then measured (n = 3 and 3, respectively, for B16-shCtrl cells and B16-SRAi cells; error bars represent standard errors; * indicates p<0.05).

We have consistently demonstrated that B16-SRAi cells show enhanced pigmentation and TRP1 expression. We then evaluated whether some intracellular signaling pathways would be disrupted after SRA knockdown (Fig 3A). We attempted to identify the common pathways that are involved in both melanogenesis or carcinogenesis and SRA signaling. The literatures search revealed that p38 MAPK [14, 21] and Notch-1 [20, 22] are indeed involved in both pathophysiological processes. Hence, we measured the activation of p38 and the cleavage of Notch1 by western blotting after inhibiting SRA. When SRA was knocked down, the constitutive p38 phosphorylation was markedly reduced, while Notch-1 cleavage was not changed (Fig 3A).

To determine whether p38 activation would lead to decreased pigmentation in B16-shCtrl cells, we incubated B16-shCtrl cells with SB202190, a p38 inhibitor (Cell Signaling), at 10 μM final concentration for 24 h and measured their melanin content (Fig 3B). The melanin content in B16-SRAi cells was 80% higher than that in B16-shCtrl cells. When B16-shCtrl cells were treated with a p38 inhibitor, the melanin content was enhanced almost to the level observed in B16-SRAi cells without p38 inhibition. Interestingly, p38 inhibition in B16-SRAi cells did not induce further increase in melanin content.

### Validation of the increased TRP1 expression and reduced p38 phosphorylation in A375 human melanoma cell line

**Fig 4:**
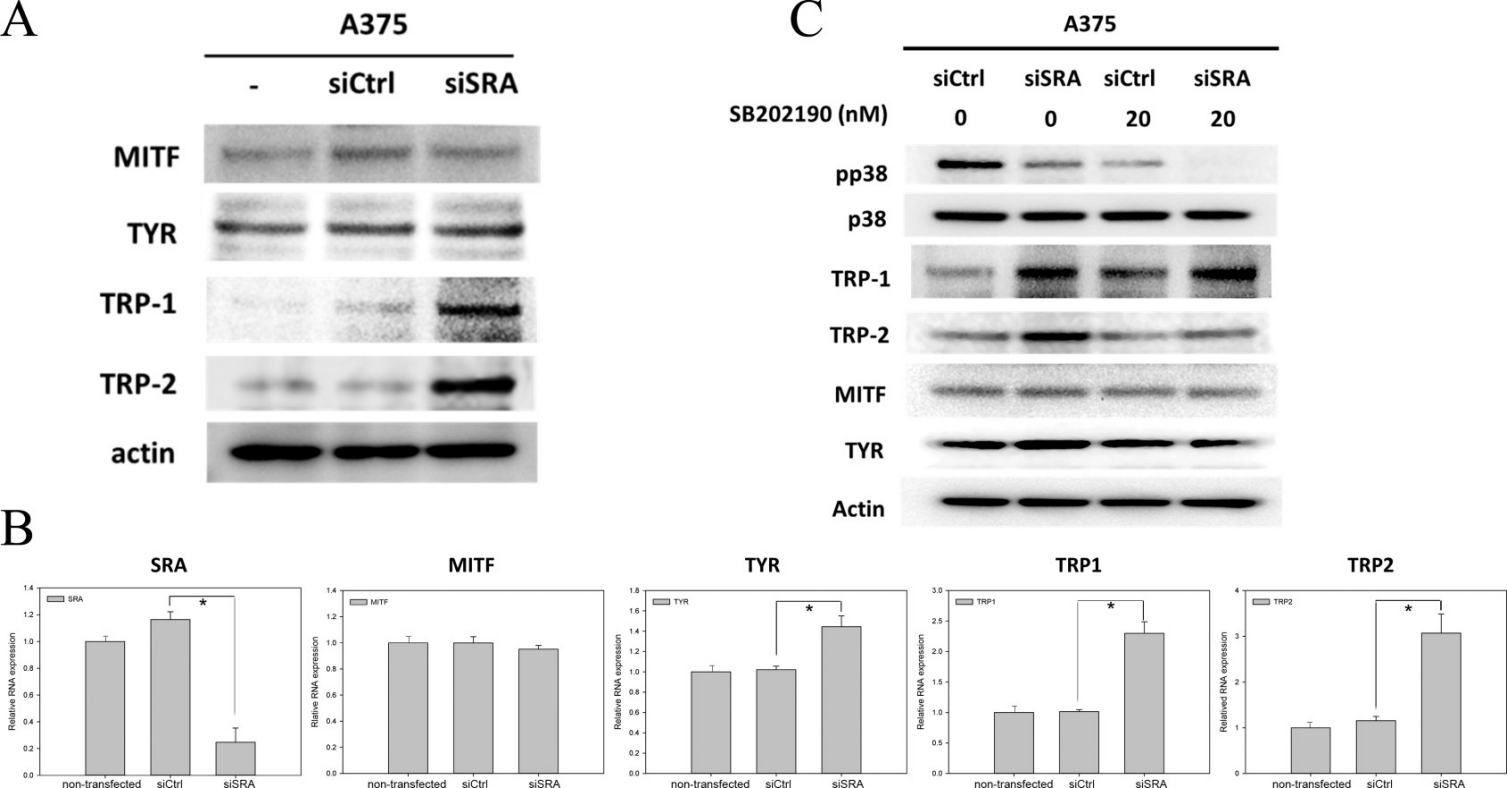
(A) RNA interference against SRA in human amelanotic A375 melanoma cells induced TRP1 and TRP2 expression. A375 cells were transfected with either siRNA against SRA (siSRA) or control sequence (siCtrl). The expression of MITF, TYR, TRP1, and TRP2 was measured by western blot. Three repeated experiments with one representative blot. (B) The A375 cells, including A375 non-transfected, A375-siSRA, and A375-siCtrl, were processed for real-time PCR to measure the expression of SRA, MITF, TYR, TRP1, and TRP2 (n = 3 and 3, respectively; error bars represent standard errors; * indicates p<0.05). (C)The expression and phosphorylation of p38 were measured in A375-siSRA and A375-siCtrl cells by western blot. To address the role of p38 in SRA activation in A375 cells, cells with or without SRA knockdown were also treated with SB202190, a p38 inhibitor, for 24 h at 10 μM. Three repeated experiments with one representative blot.

We used B16 murine melanoma cell line in the previous experiments. To investigate whether the dysregulation of melanogenic enzymes and intracellular signaling pathways in B16 cells with knocked down SRA would be recapitulated in human melanoma cell line, we used A375 cell line as it is amelanotic [23] and its p38 activation could be induced [24, 25]. A375 cells were transfected with either siRNA targeting SRA (siSRA) or a control sequence (siCtrl). The expression levels of MITF, TYR, TRP1, and TRP2 were then measured by western blot (Fig 4A). A375 cells showed an increased expression of TRP1 and TRP2 in A375-SRAi cells with non-significant changes in MITF and tyrosinase levels; this is similar to the observations in B16 cells. By using real-time PCR, increased expression of TRP1, TRP2, and TYR levels was observed, whereas no change in MITF levels was observed (Fig 4B). As we have demonstrated a decreased phosphorylation of p38 in B16-SRAi cells, we determined whether this effect could be reproduced in A375 cells (Fig 4C). The results showed that p38 was constitutively phosphorylated in A375 cells; however, its phosphorylation was decreased when siSRA was transfected into A375 cells. To determine whether p38 de-phosphorylation in A375 cells with deficient SRA would be involved in the enhanced expression of TRP1 and TRP2, A375 cells were treated with SB202190, a p38 inhibitor, followed by the measurement of the expression levels of TRP1 and TRP2 (Fig 4C). We found that SB202190 inhibited p38 phosphorylation; the expression of both TRP1 and TRP2, as expected, was significantly higher in A375-SRAi cells than in A375-siCtrl cells. Interestingly, the expression of TRP1 in A375-siCtrl cells was also induced by the treatment with the p38 inhibitor, whereas the expression of TRP2 was not. Taken together, these results indicate that the inhibition of SRA leads to the activation of TRP1 and enhances melanogenesis through the inhibition of the constitutive phosphorylation of p38.

## Discussion

In this study, we showed that SRA inhibition leads to increased pigmentations in B16 mouse and A375 human cell lines through the upregulation of TRP1 and inhibition of p38 without altering Notch 1 signaling. This is the first paper to report the regulation of human skin pigmentation through SRA. The intracellular signaling of SRA might be cell-specific, as a recent study revealed that SRA acts as a coactivator in the Notch signaling pathway in murine pre-T cells [22].

This study demonstrated that the constitutive phosphorylation of p38 in A375 cells and B16 cells was inhibited when SRA was knocked down. The inhibition of p38 activation by a p38 inhibitor also led to increased TRP1 expression, indicating that p38 activation actively reduced TRP1. The result is consistent with a report showing p38 activation could enhance pigmentations by the proteasomal degradation of tyrosinase in murine B16 cells [26]. Huang et al. reported that fenofibrate inhibits melanin synthesis through p38 MAPK activation in B16 cells [27]. However, the function of p38 in the melanogenesis in human melanocytes seems to be different from that in murine B16 cells. For example, LPS induces melanogenesis through p38 MAPK activation in human melanocytes [28]. UVB induces tyrosinase activation and melanogenesis via the ERK/p38/MITF pathway in human epidermal melanocytes [29]. In fact, the transfection of siRNA targeting Lnc-CD1D-2:1 inhibited UVB-induced p38 phosphorylation [11]. Hence, the role of p38 in melanin production might be cell-specific and thereby requires further characterization.

In this study, TRP1 and TRP2 were differentially expressed when A375 cells were treated with a p38 inhibitor. That is, the expression of TRP1, but not of TRP2, is increased in A375-siCtrl cells treated with a p38 inhibitor. It is worth noting that TRP1 and TRP2 are trafficked on distinct routes in B16 melanoma cells [30]. Therefore, targeting SRA may enhance TRP1 expression by inhibiting p38 activation independent of TRP2, which would thereby lead to enhanced pigmentation.

In B16 cells with or without SRA interference, we observed a difference in tyrosinase activity but not in the expression of tyrosine (Fig 1). In A375 cells with or without SRA interference (Fig 4), we observed a difference in tyrosinase transcript levels, but not in tyrosinase protein expression. These differences might have resulted from the functional regulation and post-transcriptional regulation of tyrosinase, respectively. For example, linoleic acid decreases the amount of tyrosinase through increased tyrosinase ubiquitination and subsequent degradation by proteasomes, a form of post-transcriptional regulation [31].

Melasma, a facial skin disease with abnormally enhanced melanin synthesis, is not uncommon in middle-aged women. It has been hypothesized that in predisposed persons, sex steroid hormones may initiate hyperpigmentation in melasma by amplifying the effects of UV on melanogenesis [32]. In fact, it has been found that estradiol [33] and diethylstilbestrol (DES) [34] upregulate tyrosinase and enhance melanin synthesis in B16 cells. On the other hand, in patients with vitiligo, which results from the decreased numbers of epidermal melanocytes, estrogen can contribute to further DNA damage in their blood lymphocytes [35]. It has also been reported that estrogen receptor 1 C/T polymorphism is associated with vitiligo [36].

In this paper, we infer that abnormal pigmentation is mediated by SRA in both B16 mouse melanoma cells and A375 human melanoma cells. However, this phenomenon might be invalid in primary human melanocytes, as these cells might be more similar to the context *in vivo* than melanoma cell lines.

In summary, we demonstrated the role of SRA, one of the lncRNAs, in the melanin pigmentation process in both human and mouse cell lines. The inhibition of the constitutive phosphorylation of p38, together with the enhancement of TRP1 expression, is important in melanogenesis and might be critically involved in the pathophysiological process of pigmentary disorders.

## Supporting information

S1 Raw Image (PDF)
