# Supplementary material for: Targeting steroid receptor RNA activator (SRA), a long non-coding RNA, enhances melanogenesis through activation of TRP1 and inhibition of p38 phosphorylation
Source: PLoS One. 2020 Aug 13;15(8):e0237577. doi: 10.1371/journal.pone.0237577 (PMC7425936; doi:10.1371/journal.pone.0237577)

1B actin

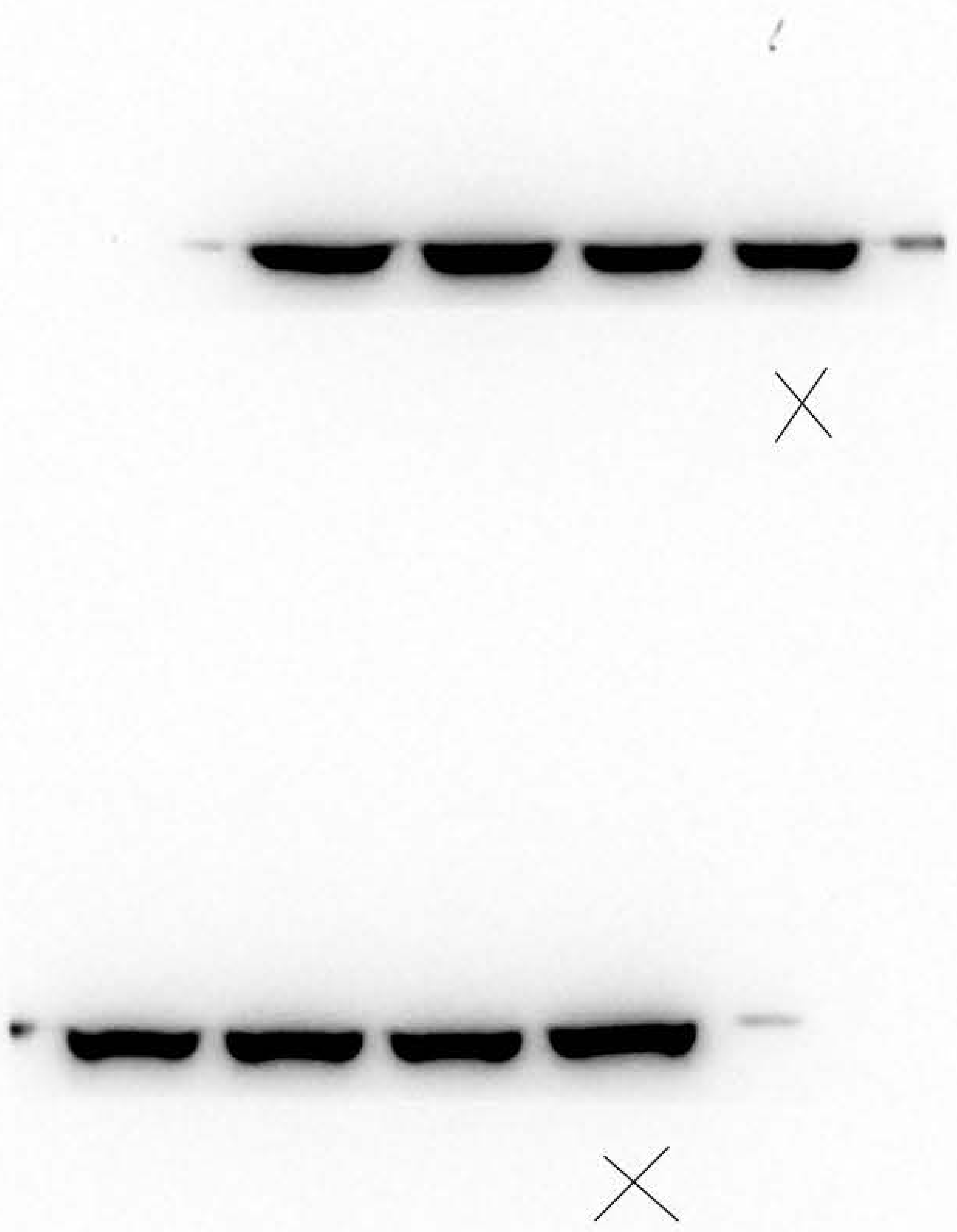

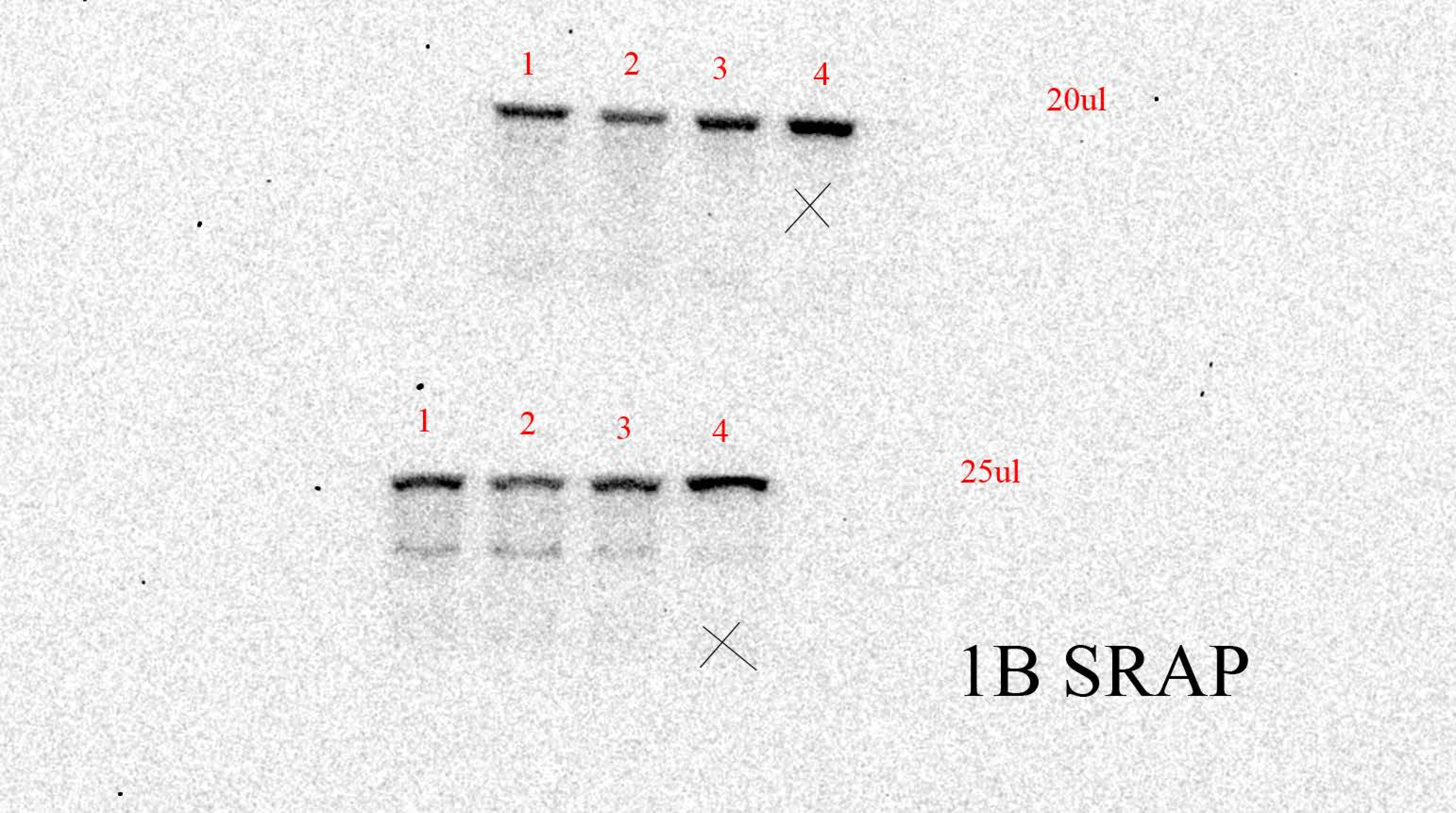
1
2
3
4
20ul
1
2
3
4
25ul
1B SRAP

1C actin

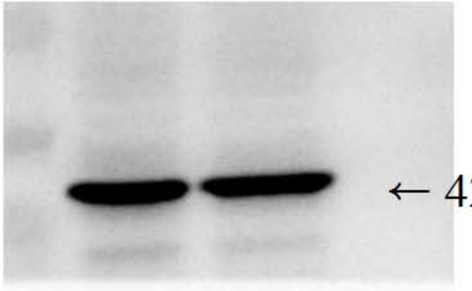

← 42 kDa

1C MITF

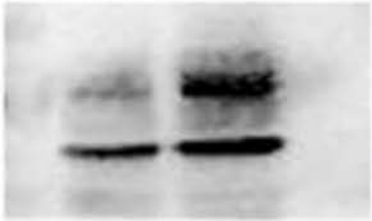

← 52 kDa

1C TRP-1

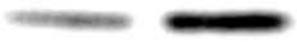

← 75 kDa

1C TRP-2

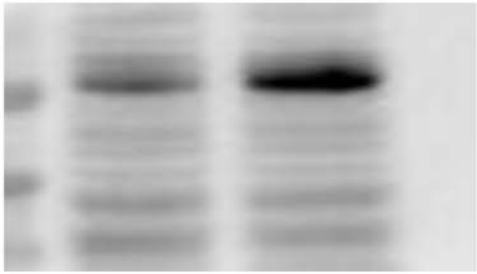

1C TYR

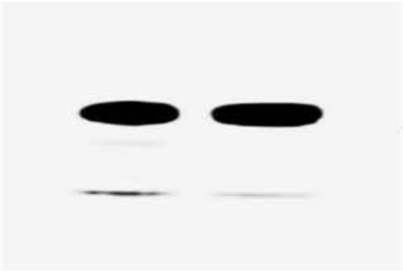

← 60 kDa

3A actin

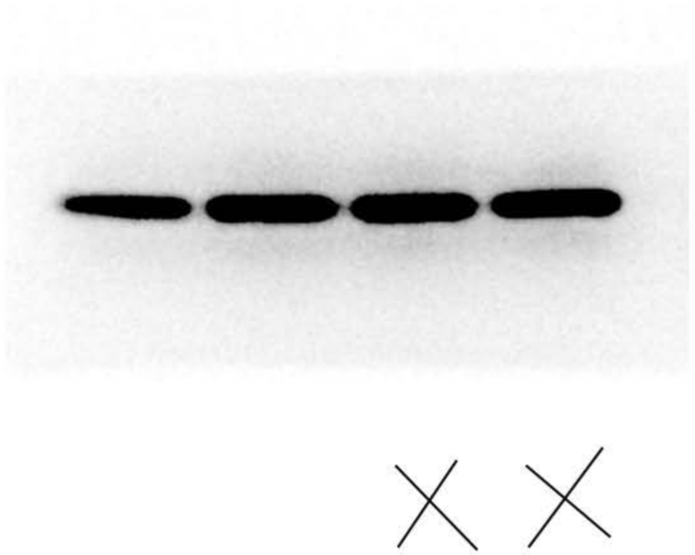

3A Notch-1

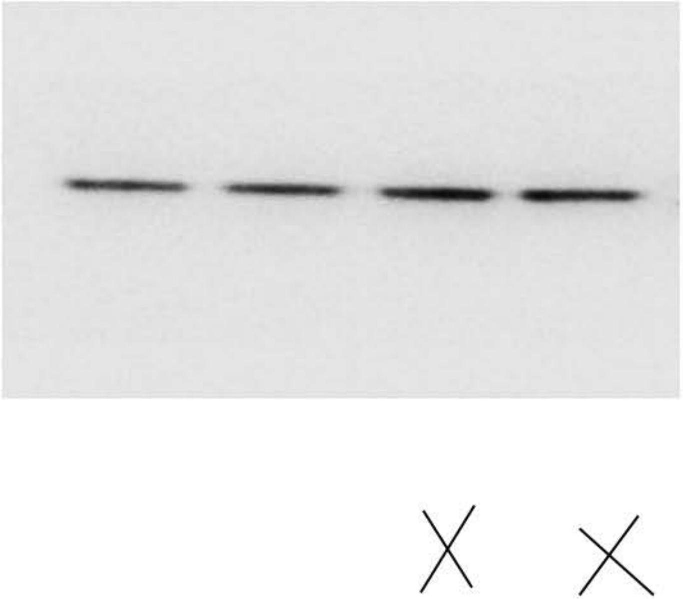

3A p38

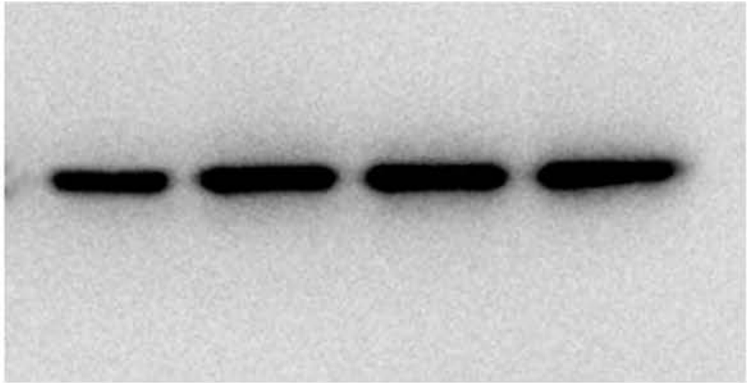

3A pp38

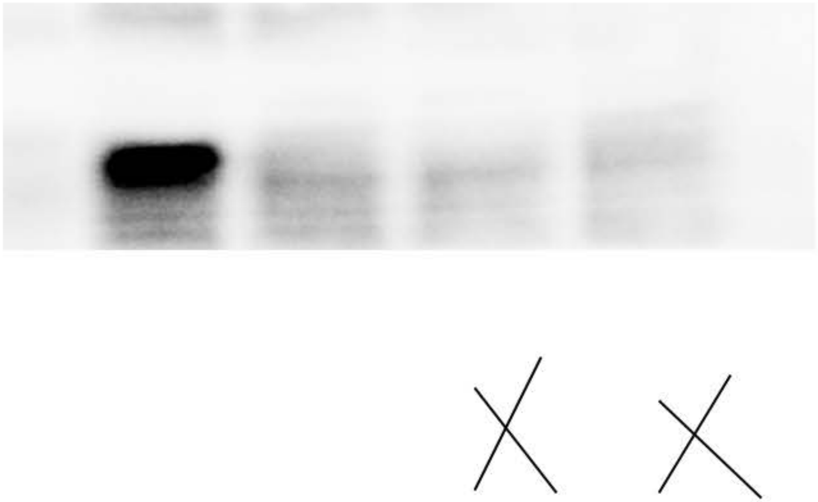

4A actin

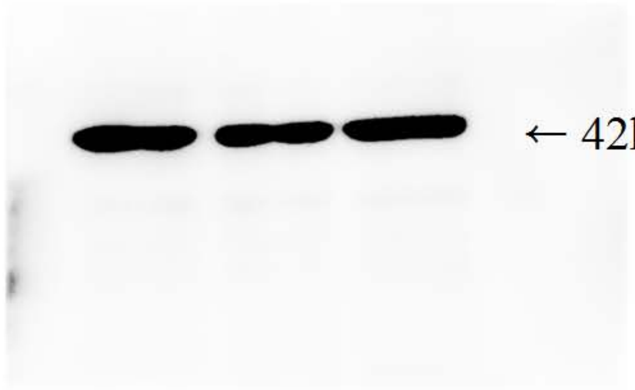

4A MITF

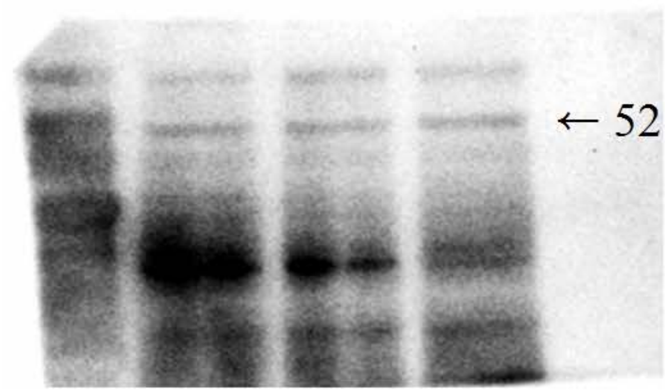

← 52 kDa

4A TRP-1

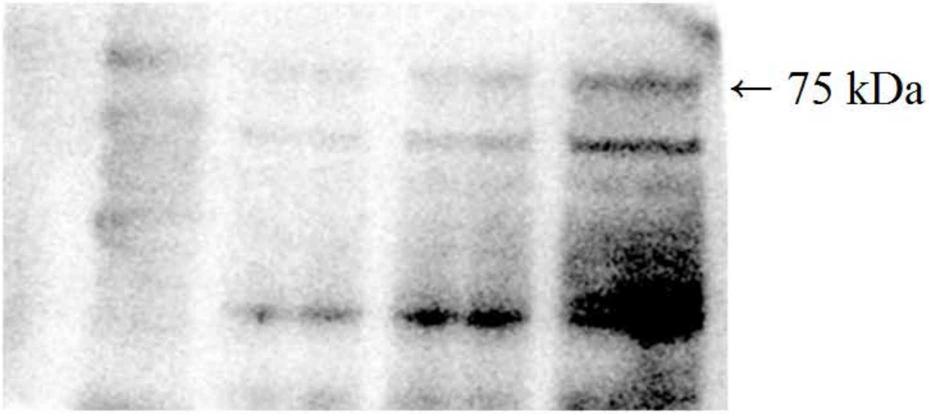

4A TRP-2

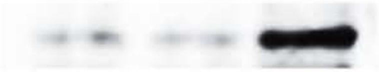

4A TYR

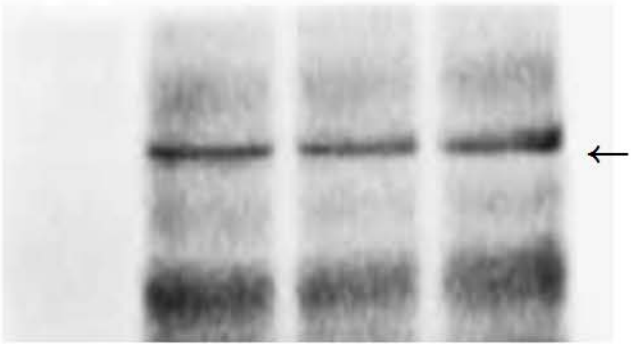

← 60 kDa

4C actin

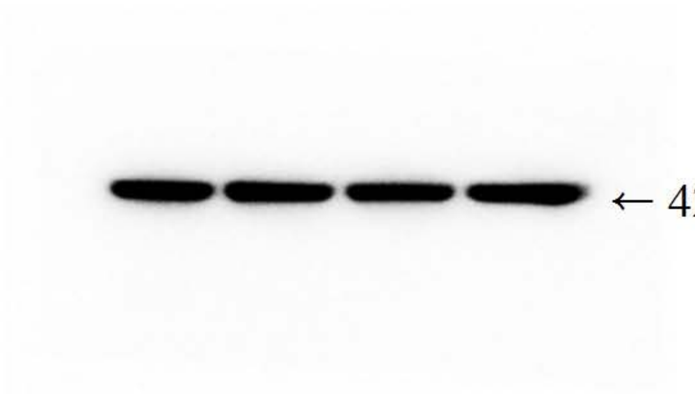

4C MITF

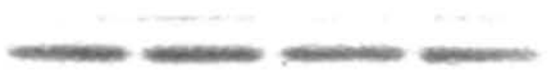

4C p38

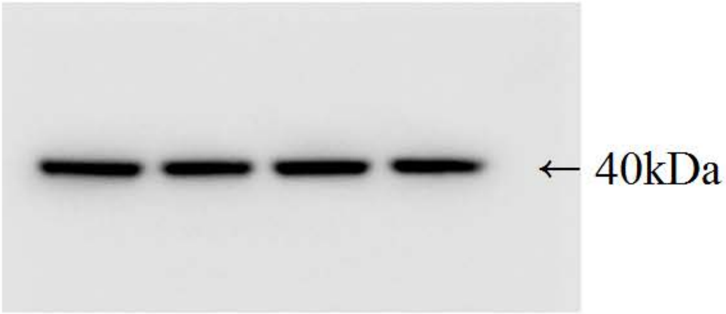

4C pp38

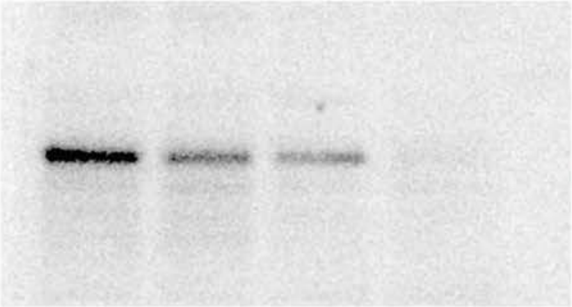

← 43 kDa

4C TRP-1

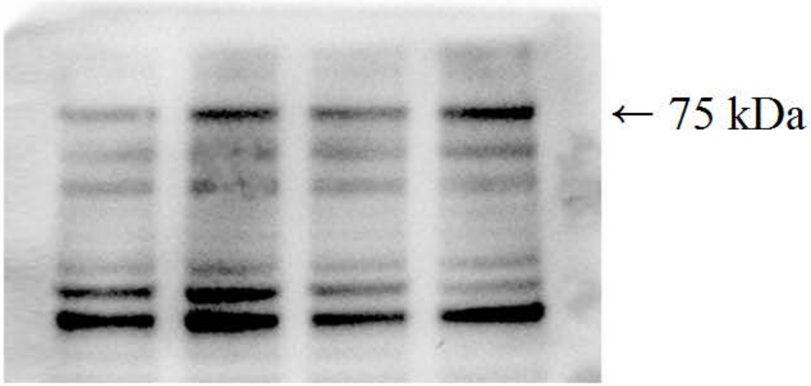

4C TRP-2

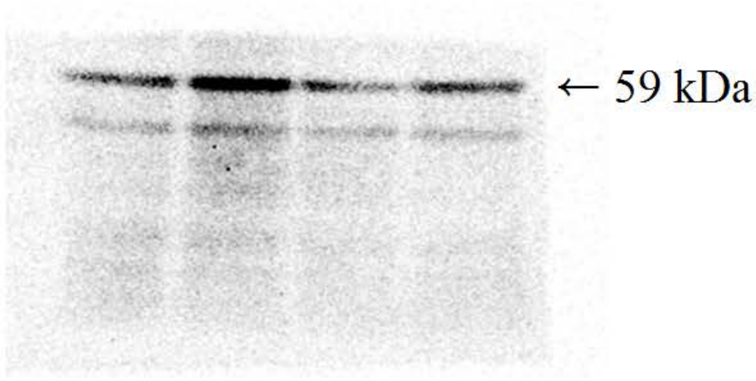

4C TYR

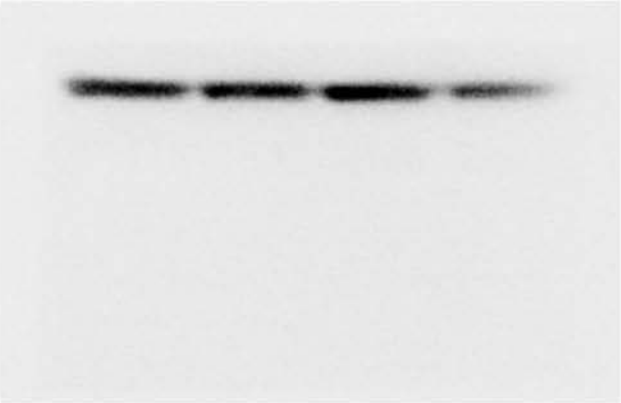

Supplement: S1 Raw Image — (PDF) [file pone.0237577.s001.pdf]
